# Recurrent gastric trichobezoar in a child

**DOI:** 10.1590/S1679-45082015AI3079

**Published:** 2015

**Authors:** Joana Morgado, Joana Gaspar, Fernanda Barros, Rui Rosado

**Affiliations:** 1 Hospital do Espírito Santo, Évora, Portugal.

**Figure 1:**
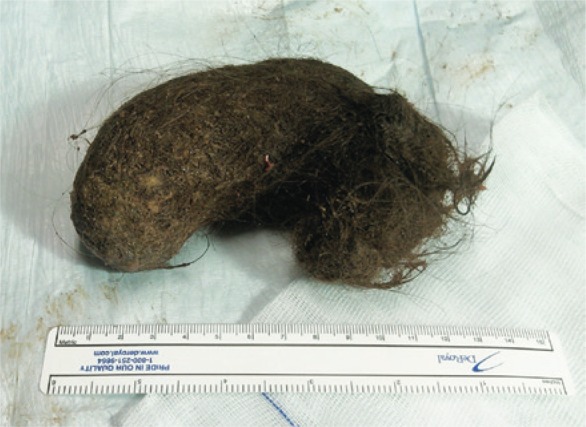
Gastric trichobezoar removed after the first laparotomy

**Figure 2:**
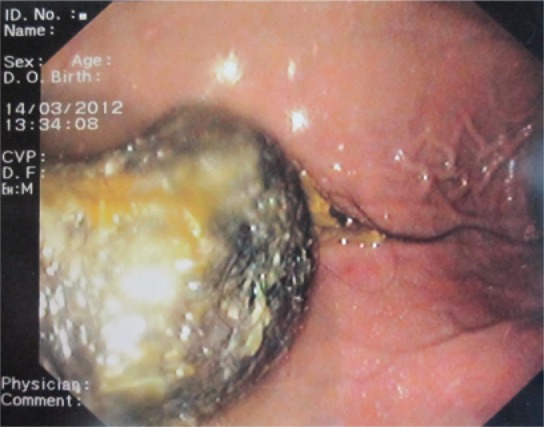
Recurrent gastric trichobezoar confirmed by upper gastrointestinal endoscopy

A 9-year-old girl with adequate growth and psychomotor development for her age was referred to our emergency service in March 2012 due to epigastralgia, postprandial vomiting, which started 2 days earlier, and a solid mobile epigastric mass of hard consistency. The patient had a history of trichophagia and, 2 years before she had been submitted to a laparotomy for removal of a large gastric trichobezoar. The upper gastrointestinal endoscopy confirmed the diagnosis of gastric trichobezoar. An anterior laparotomy was performed and a solid mass of hair of 12cm x 6cm x 4cm was removed. These dimensions were similar to those of the gastric trichobezoar removed 2 years earlier (Figure 1), in addition it presented a small tail of hair extending up to the duodenum (Figure 2). We referred the patient to a pediatric psychiatry consultation and so far she has had favorable progress without recurrent clinical or echographic signs.

Bezoars result from continuous and prolonged ingestion of indigestible materials leading to their accumulation in lumen of the digestive tube. They are classified according to their composition.^(1,2)^ Trichobezoars are composed by hair and around 90% occur in women younger than 20 years old,^(3)^ when found they can be associated with a variety of complications such as intestinal occlusion, gastric perforation, among others.^(4)^ Recurrence rate in patients who underwent surgery for gastric trichobezoar is unknown. After surgery, these patients must be referred to a psychiatric consultation and should be regularly evaluated. Patients who do not receive social and psychiatric support after the first treatment for gastric trichobezoar can present recurrence.^(5)^
