# Supplementary material for: Recurrent gastric trichobezoar in a child
Source: Einstein (Sao Paulo). 2015 Oct-Dec;13(4):640–1. doi: 10.1590/S1679-45082015AI3079 (PMC4878645; doi:10.1590/S1679-45082015AI3079)
Supplement: Translation

# Translation: Recidiva de tricobezoar gástrico em crianças

**Authors:** Joana Morgado, Joana Gaspar, Fernanda Barros, Rui Rosado

**Affiliations:** 1 Hospital do Espírito Santo, Évora, Portugal.

Paciente de 9 anos de idade, sexo feminino, com desenvolvimento estaturoponderal e psicomotor adequados para a idade, foi referenciada ao Serviço de Urgência em março de 2012 por epigastralgia, vômitos alimentares pós-prandiais com 2 dias de evolução, e massa sólida epigástrica de consistência pétrea e móvel. A paciente tinha história de tricofagia e, 2 anos antes, tinha sido submetida à laparotomia para remoção de volumoso tricobezoar gástrico. A endoscopia digestiva alta confirmou a hipótese de diagnóstico de tricobezoar gástrico. Foi realizada nova laparotomia anterior e retirada massa sólida de cabelos com 12cm x 6cm x 4cm, dimensões estas semelhantes às do tricobezoar gástrico removido 2 anos antes (Figura 1), e pequena cauda de cabelos que se estendia pelo duodeno (Figura 2). Essa paciente foi, então, referenciada a uma consulta de psiquiatria da infância e da adolescência, apresentando, até a data, evolução favorável, sem evidência clínica ou ecográfica de recidiva.

**Figura 1:** Tricobezoar gástrico removido após primeira laparotomia

**Figura 2:** Recidiva de tricobezoar gástrico visualizado em endoscopia digestiva alta

Os bezoares resultam da ingestão continuada e prolongada de materiais não digeridos, levando à sua acumulação no lúmen do tubo digestivo. São classificados de acordo com sua composição.^(1,2)^ Os tricobezoares são compostos por cabelo ou pelo, ocorrendo cerca de 90% em doentes do sexo feminino com idade inferior a 20 anos.^(3)^ Quando presentes, podem estar associados a inúmeras complicações, como oclusão intestinal, perfuração gástrica ou outras.^(4)^ Na literatura, não é conhecida a taxa de recorrência em pacientes operados a tricobezoares gástricos. Após cirurgia, esses pacientes devem ser referenciados a consulta de psiquiatria e serem avaliados regularmente. Pacientes que não recebem apoio social e psiquiátrico após o primeiro tratamento podem sofrer recidiva.^(5)^
